# Influence of Changing Weather on Old and New Maize Hybrids: A Case Study in Romania

**DOI:** 10.3390/plants13233322

**Published:** 2024-11-27

**Authors:** Roxana Elena Călugăr, Andrei Varga, Carmen Daniela Vana, Loredana Ancuța Ceclan, Ionuț Racz, Felicia Chețan, Alina Șimon, Călin Popa, Nicolae Tritean, Florin Russu, Alexandru Bogdan Ghețe, Leon Muntean

**Affiliations:** 1 Laboratory of Maize Breeding, Agricultural Research and Development Station Turda, 401100 Turda, Romania; roxana.calugar@scdaturda.ro (R.E.C.); andrei.varga@scdaturda.ro (A.V.); carmend.vana@yahoo.com (C.D.V.); ancaceclan@gmail.com (L.A.C.); ionut.racz@usamvcluj.ro (I.R.); calin.popa@scdaturda.ro (C.P.); 2 Department of Genetics and Plant Breeding, Faculty of Agriculture, University of Agricultural Sciences and Veterinary Medicine Cluj-Napoca, 400372 Cluj-Napoca, Romania; leon.muntean@usamvcluj.ro; 3 Laboratory of Technology and Mechanization, Agricultural Research and Development Station Turda, 401100 Turda, Romania; felice_fely@yahoo.com (F.C.); alina.simon@scdaturda.ro (A.Ș.); 4 Department of Technical and Soil Sciences, Faculty of Agriculture, University of Agricultural Sciences and Veterinary Medicine Cluj-Napoca, 400372 Cluj-Napoca, Romania; alexandru.ghete@usamvcluj.ro

**Keywords:** maize, drought, heat, water stress, ASI, yield

## Abstract

Maize is affected by drought and heat, abiotic stress factors that have been encountered more often in recent years in various parts of Europe. In the area of Turda, Romania, extreme temperatures and heat waves combined with an uneven distribution of precipitation have been recorded that had an unfavorable influence on the maize crop. In this study, the ASI (anthesis-to-silking interval), yield, and stability of 35 old and new maize hybrids created at the Agricultural Research and Development Station Turda were studied under drought and heat conditions. An increase in temperature was observed during vegetative growth and grain filling, and rainfall was deficient during and after flowering. These conditions had a negative influence on ASI, grain filling, and, indirectly, yield, which varied significantly during the seven experimental years. The five newest hybrids (Turda335, Turda2020, Turda380, Sur18/399, and HST148) stood out, with average yields of over 8400 kg ha^−1^ in unfavorable years and over 15–16,000 kg ha^−1^ under favorable conditions. They generally outperformed the mean by 29–33%. In contrast, the old hybrids achieved yields up to 22% lower than the experimental mean. Yield was 43.1% lower in 2022 and 31.8% lower in 2023 compared to the best year (2021).

## 1. Introduction

Maize is one of the most important crops for humanity, but its production is strongly affected by stress factors such as drought and heat. Drought is one of the most important abiotic stress factors and affects crop productivity for about a third of the world’s population [1].

In recent years, a trend of increasing average temperatures has been observed worldwide, and in Europe, the temperature variation in a year was greater than 1 °C [2]. Several extreme summertime droughts have been reported since the beginning of the 21st century [3] in different parts of the continent [4].

In Romania, according to the National Meteorology Administration (NMA) [5], the warmest years (from 1961 to 2023) were 2023, 2019, 2020, 2022, 2015, and 2018. In recent years, there have been some notable deviations from the multi-year average as follows: the warmest January in the history of meteorological measurements was in 2023, with the maximum temperature exceeded at 72 out of 129 weather stations; in July 2022, maximum temperatures were exceeded (the highest deviation was 2.7 °C) at several weather stations, including in Turda; and in 2022 and 2023, the highest average temperatures in August were recorded. An upward trend in temperatures was also observed during the months critical for the maize crop, June, July, and August, when anthesis and grain formation are achieved. The years 2019 and 2022 are noteworthy, with deviations of 2.59 °C and 2.88 °C, respectively [2]. If these high temperatures are combined with a lack of rainfall, yield can be greatly affected.

In the southern part of Romania, in particular, long-lasting droughts have been repeatedly reported [6,7,8,9,10], with some areas being desertified [8,11,12,13,14]. Heat waves have also been recorded, with their frequency [15,16,17,18] and duration increasing after 2000. In June 2019, a heat wave lasted for 10 days and affected more than 90% of the country [18]. In Romania, the largest areas are affected by extreme drought between June and August (over 5 mil ha) [10]. The drought of the last decades has led to a decrease in groundwater resources in the country [19,20].

After studying more than 70 references related to maize, Sánchez et al. [21] concluded that the maximum temperatures for achieving anthesis, grain filling, and good growth and development of the whole plant are 37.3 °C, 36 °C, and 42 °C, respectively [21]. The normal functioning of the plant is reduced; however, even at temperatures as high as 35 °C, it is disrupted by physiological processes. If these limits are exceeded, the proper functioning of physiological processes is negatively influenced and the viability of the pollen decreases [22], resulting in lower grain yield.

Silk growth is much more sensitive to a lack of water and increased temperature, and the phenomenon of protandry is accentuated due to the apical tassel dominance of maize and better tolerance to the development of the tassel and the shedding of pollen. These differences between flowering and silking can result in yield losses of 20–50% [23,24]. The flowering period is considered the most vulnerable to heat stress [25,26], and some breeding programs specifically aim to reduce the ASI by using germplasm from arid areas [27]. Fertilization is also disturbed, and grain filling may be affected, with potential increases in grain abortion and the percentage of sterile plants. In cases of extreme drought and in some more sensitive genotypes, the drying of young leaves and tassels can be observed. Drought also affects the development of the vegetative parts, namely, the shoot and root length [28], plant and ear height [23], and husk size. Following an analysis of 144 studies on the effects of drought on maize, it was concluded that production losses of approximately 39% are due to this stress factor [29].

Some forecasts indicate that drought-affected areas will expand; therefore, breeding genotypes with increased tolerance is necessary [30,31,32]. Maize breeding for tolerance to drought stress starts with obtaining superior parental forms and identifying the sources that can be used in the breeding processes to improve other genotypes [24,25,30,31,32,33].

The aim of this study was to evaluate the yield differences between 35 hybrids and yield stability in relation to environmental conditions. The identification of genotypes with the lowest possible ASI was also pursued, as it is an indicator of tolerance to abiotic stress during the anthesis period. The number of days between sowing and the flowering phase was also monitored in order to differentiate the necessary period between the two phases, depending on the meteorological conditions in each of the seven analyzed years.

## 2. Results

### 2.1. Variation in the Climatic Conditions of the Experimental Years

An increase in average temperatures compared to the 60-year average has been observed over the years in the studied area. These increases are of particular importance for maize, especially in the summer months, when pollination and grain filling take place. In all the analyzed years (2017–2023), summer temperatures exceeded the multi-year average. The distribution of precipitation was very uneven during the growing season and either exceeded the average values or was well below the optimal threshold. Although in some years, the level of precipitation was consistent with the specifics of the area, its uneven distribution was unfavorable to some agricultural crops, including maize.

Large differences in temperature and precipitation were observed between years and within the same month (e.g., rainfall was recorded but only in the last days of each month); therefore, for a better interpretation of the phenomena encountered, each month was divided into three 10-day periods.

**Figure 1:**
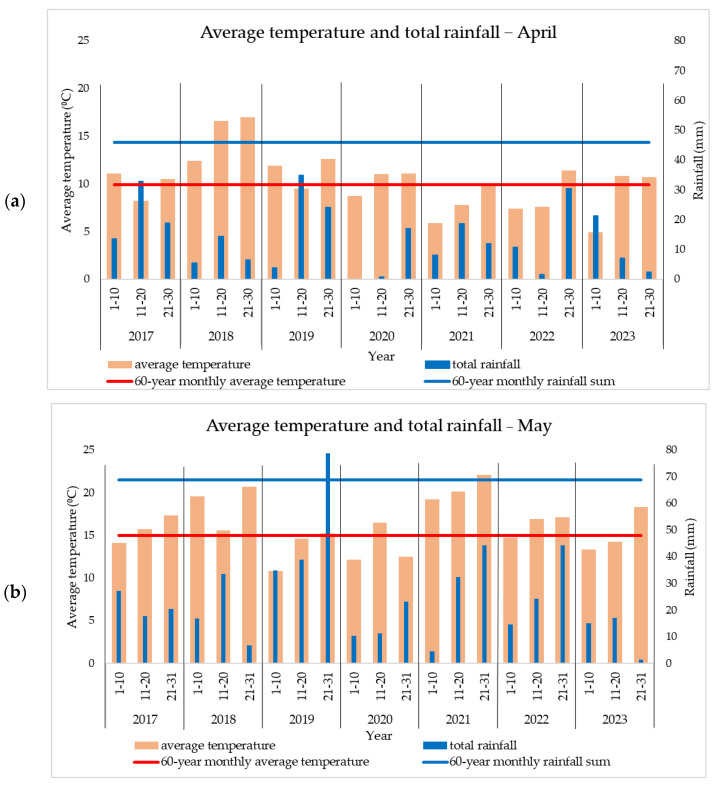
Average temperature and rainfall during sowing and crop emergence in (**a**) April and (**b**) May.

**Figure 2:**
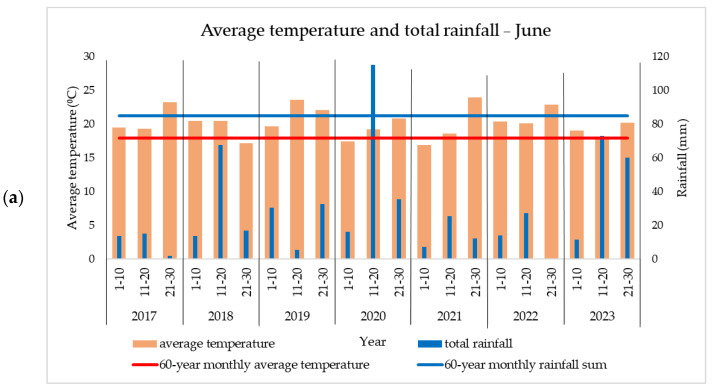

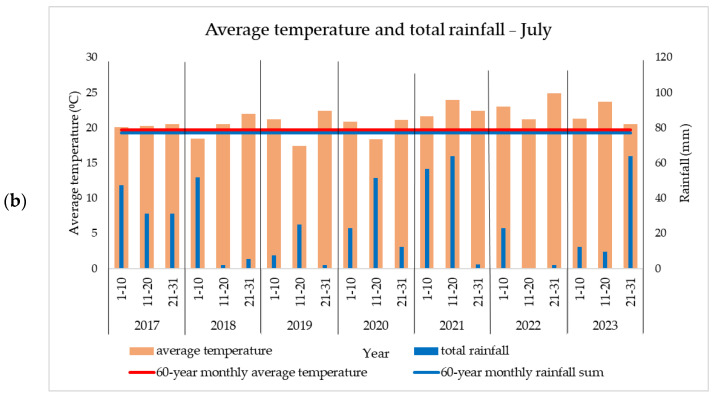
Average temperature and rainfall before and after pollination in (**a**) June and (**b**) July.

In 2017, a rainfall level appropriate to the area was recorded during sowing and crop emergence (May) (Figure 1), but in June, high temperatures (+2.7 °C compared to the average) and lack of rainfall (−54.2 mm) affected the crop (Figure 2). Even though the rainfall level was higher in July (110.2 mm/month) (Table 1), the yield was already negatively affected. August was unusually warm, with temperatures over 32 °C recorded on 12 days (Table 2).

Even though higher temperatures and low rainfall were recorded in April and May 2018 (Figure 1), the soil water reserve due to the precipitation recorded in the first months of the year favored good crop emergence. For this year, the largest deviations in the average monthly temperature were recorded as follows: +5.4 °C in April and +3.7 °C in May. Weather conditions from June to August were favorable for maize cultivation.

In 2019, rainfall exceeded the area-appropriate value in April and May (Figure 1), but drought occurred in the following months. In June, the average temperatures exceeded the multi-year average by 3.9 °C, and in July, although the temperatures were normal, the rainfall was deficient (−42.1 mm) (Table 1). In August, temperatures over 32 °C were recorded for 9 days (Table 2).

In 2020, both temperature and rainfall had values close to those appropriate to the area, positively influencing the maize crop.

The year 2021 was one of the most favorable for maize cultivation due to normal or above-normal temperatures and above-average rainfall. In July, temperatures over 32 °C were recorded on 11 days (Table 2), and the 123.1 mm of rainfall (Table 1) favored pollination and thus indirectly contributed to large harvests.

**Figure 3:**
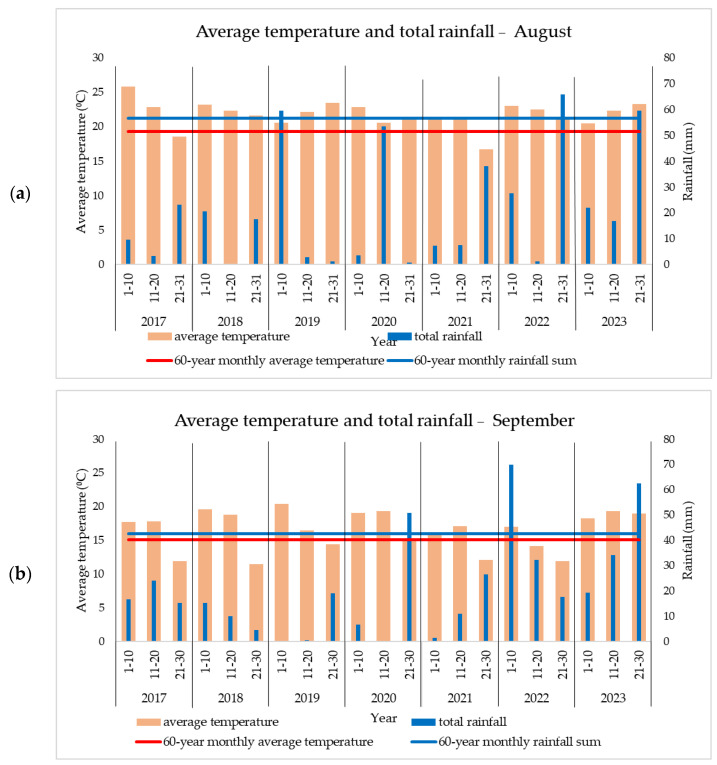
Average temperature and rainfall during grain formation and harvest in (**a**) August and (**b**) September.

In 2022, a year with drought problems, rainfall was recorded in the latter part of April and in May (Figure 1). This rainfall followed a period of precipitation deficit that occurred in the first three months of the year and in early April. The year was atypical with respect to maximum temperatures as it was the first with temperatures above 35 °C in June, July, and August (Table 2). During this period, three heat waves were recorded. Rainfall was also very low as follows: −43 mm in June and −53 mm in July. The rainfall recorded in the latter part of August and early September (Figure 3), which came after a prolonged period of drought, had only negative effects, such as the emergence of pathogens and early plant senescence (BBCH 97).

In 2023, although the rainfall in the summer months was normal or even above the area’s average, the high temperatures had a negative effect on the crop. In August, temperatures over 32 °C were recorded on 13 days (Table 2), while September was the warmest in recent years (Figure 3), with temperatures +3.8 °C compared to the multi-year average.

The increased thermal values in July were associated with a rainfall distribution that was uneven in both the number of days with precipitation and amount (Table 1). The lowest number of rainy days was recorded in 2021 and 2022, but the amount of precipitation was very different. Although there were 12 consecutive days without rain in 2021 (4–15 July), the heavy rainfall of the previous days and the days that followed helped the plants to develop normally. In 2022, there were 17 consecutive days without rainfall, the amount of rain was also much lower, and the temperatures were higher. In 2018 and 2023, the same number of rainy days and the same amount of precipitation were recorded, but in the former, precipitation was recorded in the first part of July, and in the latter, it occurred towards the end of July, when the crop was already affected.

Heat represents the maximum number of daily air temperatures that exceed the critical biological threshold of 32 °C and is expressed by the intensity (sum of temperature degrees above 32 °C) and the duration (number of days) of the phenomenon [34]. Temperatures exceeding the threshold of 32 °C or even 35 °C were reported in June and July 2017, 2019, 2021, 2022, and 2023 (Table 2).

### 2.2. Reaction of Hybrids at BBCH65

**Figure 4:**
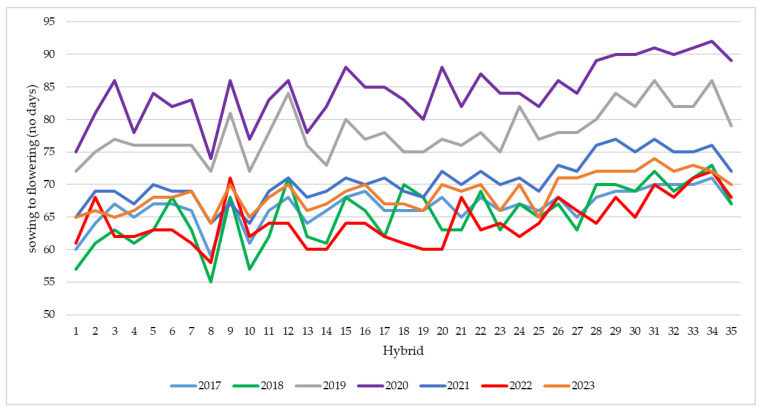
Number of days between sowing and flowering (BBCH65).

The efficiency with which sunlight is used in the physiological processes of the plant is closely related to the duration of time in which the canopy can intercept this light. Declining sunshine hours reduce crop yield by weakening photosynthesis [35,36,37,38,39,40]. Sunshine is of particular importance in the pre-flowering period, influencing ear formation, and after pollination to avoid grain abortion [41]. In May and June 2020, a reduced number of sunshine hours were recorded, thus extending the period between sowing and flowering to an average of 84 days (Table 3). All studied hybrids needed a longer period to reach flowering in 2020, with the next longest period being recorded in 2019 (Figure 4). For most hybrids, the lowest number of days between sowing and flowering was noted in 2022, but for some of the early hybrids (HD115, HS105, HS105A, Turda100, Turda199, Turda-Mold188, and Turda165), the lowest number of days was in 2018.

In May 2018 and 2022, the highest GDD value and the most hours of sunshine were calculated, causing a reduction in the number of days between sowing and flowering.

**Figure 5:**
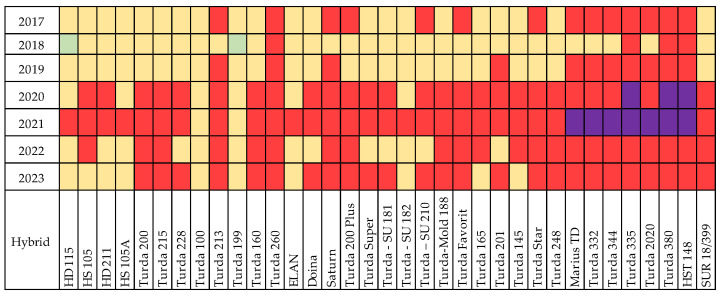
Anthesis period: green = June; yellow = 1–10 July; red = 11–20 July; purple = 21–31 July.

For the years 2020 to 2023, flowering (BBCH65) occurred mostly between 10 and 21 July (Figure 5), a period with high temperatures. In 2020 and 2021, rainfall was recorded, with pollination being completed in optimal conditions. In 2022 and 2023, the lack of rainfall during anthesis favored the occurrence of protandry and poor pollination. Hybrids HD115, Turda100, and Turda199 reached the BBCH65 phase much earlier regardless of the year (Figure 4 and Figure 5), but the shorter vegetation period was closely related to a lower yield.

**Figure 6:**
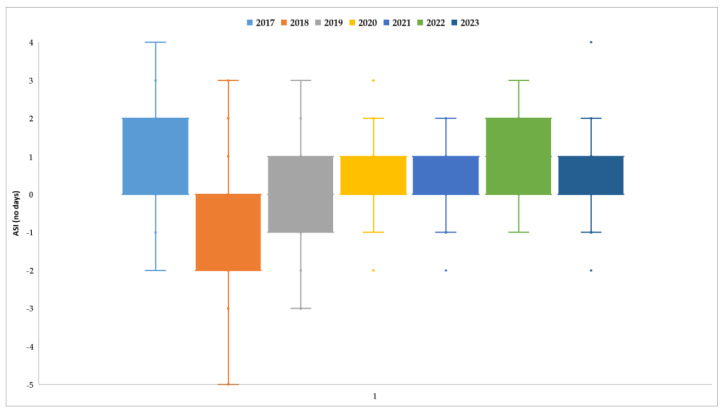
Anthesis-to-silking interval (number of days).

Protandry was observed mostly in older hybrids and especially in years with higher temperatures. The highest ASI values (4–5 days) were recorded in 2017, while the lowest values were noted in 2021 (Figure 6). In 2018, the highest frequency of protogyny was noted, in 15 out of the 35 hybrids, while another 13 had flowering–silking coincidence. For most hybrids, pollination occurred between 1 and 10 July, when the average temperature was 18.5 °C and rainfall totaled 51.9 mm.

The most cases of protandry (18) were reported in 2022. Rain in the first week of July (21.3 mm) had a positive effect on pollen release and silk emergence. For the hybrids that flowered before the rains, protandry was strongly manifested. In the breeding process of the new hybrids at ARDS Turda, the best flowering–silking coincidence was taken into account. The hybrids registered in the last 20 years (Doina, Turda165, Turda Star, Turda344, Turda335, Turda2020, and Turda380) have a good coincidence, regardless of the climatic conditions.

### 2.3. The Yield Obtained in the Conditions of the Experimental Years

In 2018, 2020, and 2021, the yield exceeded the average, but in the other years, the differences were very significantly negative. The lowest yield was obtained in 2022, with a difference of −2854 kg ha^−1^. Significant negative differences also occurred in 2023, with average yield values 1360 kg ha^−1^ lower than the multi-year average (Table 4).

The genetic progress achieved in the creation of maize hybrids can be observed, with the newest creations exceeding the experimental average with very significant differences of up to 2895 kg ha^−1^ (Table 5). The superior yields obtained by the newest maize hybrids, Turda332, Turda344, Turda335, Turda2020, Turda 380, HST148, and SUR18/399, are due to both their high yield capacity and the adaptive heterosis they exhibit. All six hybrids are characterized by a well-developed leaf system, erect or semi-erect leaves, good flowering–silking coincidence, and a low number of sterile plants [42,43,44]. Turda380 is also a prolific hybrid and produces a low number of sterile plants even in unfavorable years. To withstand heat stress, some plants can reduce conductance by curling their leaves to avoid water loss through evapotranspiration [45], a behavior noted in recent hybrids, particularly in Turda 380. A higher tolerance to unfavorable conditions was also observed in the parental genotypes of the previously mentioned hybrids.

**Figure 7:**
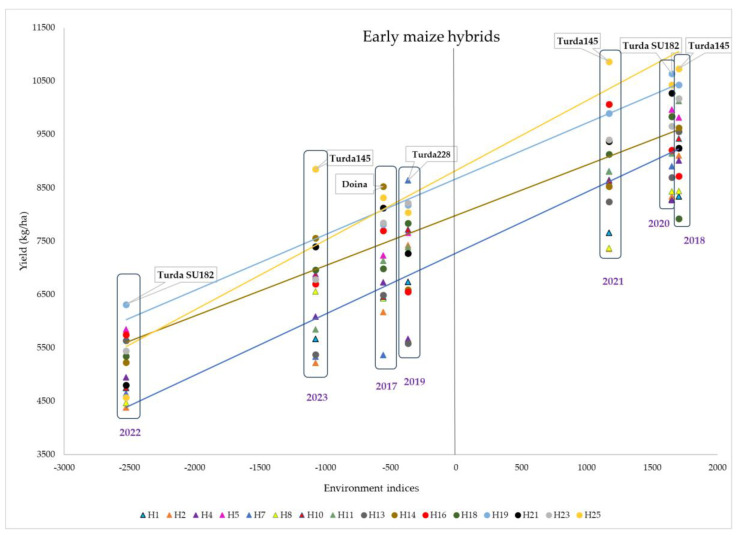
Yield stability of early maize hybrids.

**Figure 8:**
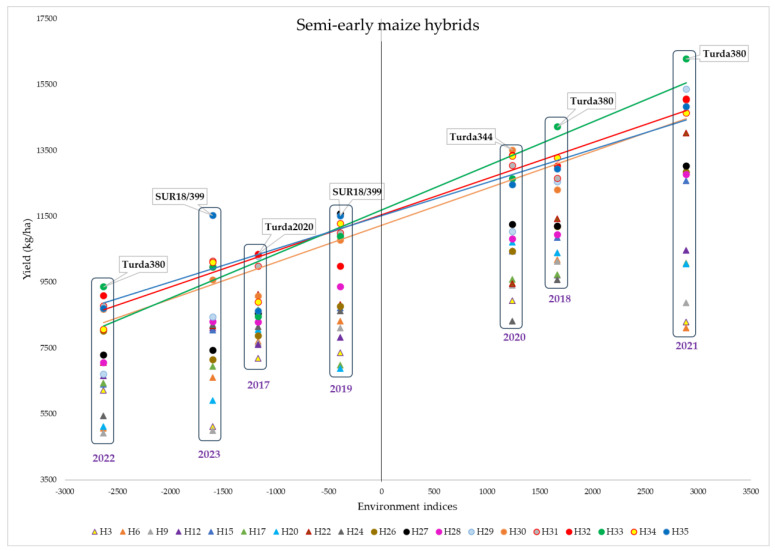
Yield stability of semi-early maize hybrids.

The hybrids were analyzed in terms of yield stability over the 7 years, and the genotypes were divided into two groups, depending on the maturity period, that is, early (Figure 7) and semi-early (Figure 8). Each point on the graph represents the intersection of the environmental indices (calculated by the difference between the mean yield of each year and the overall average) and the yield obtained by each of the hybrids in that year.

Of the early hybrids group, Turda145 had the highest yield in 2023, 2021, and 2018 but was strongly influenced by unfavorable conditions. The hybrid Turda SU182 had good stability, with a higher yield even in unfavorable years (Figure 7).

Semi-early hybrids are generally more recent creations with a genetic base improved over a longer period of time to be better adapted to stress factors and have a higher yield capacity. Better stability was observed for the hybrids Turda344, Turda2020, Turda335, Turda380, HST148, and SUR18/399 (Figure 8).

The Turda380 hybrid achieved the highest yield in the experimental system in 2018, 2021, and 2022 and was among the highest-yielding genotypes in the remaining years as well. This hybrid is characterized by a high plant height, uniform and high ear insertion, erect leaf bearing, good grain water loss capacity [42], and a good tolerance to high densities [45]. Hybrid SUR18-399 was among the most productive in all the unfavorable years.

Comparing the two groups, it can be noted that the unfavorable years had the same order (2022, 2023, 2017, and 2019); however, the yield difference between 2022 and 2023 was smaller for the semi-early hybrids, indicating a greater sensitivity of the early genotypes to the drought conditions in 2022. For the early hybrids, the most productive years were 2018, 2020, and 2021. For the semi-early hybrids, however, the best year was 2021, when the highest yields were obtained, indicating its clearly superior conditions in terms of favorability even compared to the other two good years, namely, 2018 and 2020.

## 3. Discussion

### 3.1. Drought and Heat Effect on Maize

Drought and heat have a negative influence on morphological, biochemical, and physiological processes in maize plants. These stress factors can cause yield reduction, reduced seed germination, withering and drying of leaves, tassel drying, delayed silking, poor pollination, incomplete cobs, defective husk growth (which favors attack by some pests), reduction in all vegetative parts of the plant, an increase in the number of sterile plants, reduced grain weight, influence on grain quality, premature senescence of the plants, etc. [46,47,48,49]. All these were observed in the maize breeding fields under drought conditions, especially in 2022, when these symptoms were more frequent.

Edmeades [50] describes the appearance of the negative effects of drought stress in three stages as follows: 1. before anthesis, affecting meiosis; 2. delayed appearance of stigmas or shortening of the optimal pollination period, resulting in defective pollination; and 3. abortion of the grains a few days after fertilization. During the flowering phase, if temperatures exceed 30 °C and are accompanied by water stress, they are harmful to the optimal fecundation process [51]. This period is the most susceptible to drought damage [48,49], with a water deficit resulting in delayed ear growth and silking [52]. Pollen can be damaged or lose viability at temperatures above 32 °C [49]. The largest yield losses were reported in 2022, when the dry conditions occurred during the flowering phase.

High temperatures can affect pollen viability, but considering that pollen is released for several days and generally in the morning, before the temperatures rise excessively [53], if the water reserve in the soil is adequate for the needs of the plant, optimal pollination can be achieved even under extreme temperature conditions. The problem arises from the delayed silking and the shortening of the optimal pollination period.

Water deficit increases the ASI and negatively affects pollen release and silk development, thus resulting in a yield reduction of approximately 3–8% [54]. Unpollinated silk continues to elongate for about 10 days, but due to the delayed silking, little or no viable pollen remains [55].

A water deficit from a few days before silking to about 25 days after can significantly reduce the number of grains per ear and, indirectly, the yield [56,57,58]. In 2022, there were several days with high temperatures and without precipitation during the pollination period, resulting in a lower yield. In 2021, although there were 12 consecutive days without precipitation in July, these were preceded and followed by rainy days, thus ensuring the necessary water. The results obtained from heat stress testing under irrigated versus non-irrigated conditions indicate that if the water available to the plant is sufficient, high temperatures have a minimal negative effect on yield [59]. Our data confirm that high temperatures in combination with abundant rainfall and optimal distribution during the pollination period result in higher yield.

The highest yield was obtained when both temperatures and rainfall were normal or high (in 2018, 2020, and 2021). When pollination took place under hot and dry conditions (the last two years), the effect on the yield was negative. After reviewing several studies, Daryanto et al. [29] stated that when the humidity was reduced by about 40%, a decrease in yield of about 39% was observed. Compared to 2021, a favorable year for maize cultivation, the decreases in yield in the next two years were close to the reported values of 43.1% in 2022 and 31.8% in 2023.

### 3.2. Old vs. New Hybrids

The older hybrids were created, tested, and cultivated during a time when the average temperature of the summer months was lower. In the first two decades after the establishment of ARDS Turda (1957–1977), the average temperature in July was about 19.4 °C, with an annual average of 8.9 °C [60]. The 2017–2023 temperature average for July was 20.8 °C, and the annual average was 10.7 °C.

Protandry was observed, especially in older hybrids, even under favorable environmental conditions, indicating their reduced ability to adapt to increasing temperatures. Hybrids registered after 2000 show very good coincidence or even protogyny during the pollination period. This aspect is in agreement with the findings of Barker et al. [61], who attribute the sensitivity of older genotypes to the fact that they did not benefit from such a long period of germplasm improvement. Elmore [62] also indicates a reduction in the ASI for hybrids created in recent decades, while for the older hybrids, the water stress resulted in delayed silking. In recent years, several authors [63,64,65] have reported a rainfall deficit and increasing temperatures during anthesis, leading to a delay in flowering–silking of 3–4 days [66,67], but delays of up to 8 days [68] have also been noted under stress conditions. In our study, a maximum ASI of 4–5 days was noted.

### 3.3. Future Prospects

It is essential to obtain high-yielding and stable maize hybrids, especially considering the changing climate, but this is difficult due to the high complexity of traits and large number of genes involved [69,70,71]. Knowing the reactions of the genotypes to stress factors can provide valuable information for future breeding programs. It is necessary to create new genotypes adapted to recent climate conditions that can tolerate extreme temperatures and a lack of precipitation, but cultivation technologies must also be improved so as to preserve soil moisture.

Testing of the newest hybrids will continue under additional stress conditions (variable sowing densities, minimum tillage, different sowing seasons, and artificial infestation with pathogens). The five newest creations will be cultivated in the following years at other research stations, taking into account the different pedoclimatic conditions compared to the local ones.

A future research direction also includes breeding new genotypes with erect leaf bearing, the ability to contract the leaves to avoid evapotranspiration, a low ASI, well-covered ears, and a reduced number of sterile plants. Even if a high yield is desirable, stability is of particular importance, so the breeding program aims to obtain not just a record yield under optimal conditions but also a good and stable yield, regardless of the environmental conditions.

## 4. Materials and Methods

### 4.1. Biological Material

The 35 maize hybrids (Table S1) created at ARDS Turda were tested during 2017–2023 for yield and yield stability under drought and heat stress. The anthesis (BBCH 65) and ASI were also monitored, depending on the climatic conditions of each year.

Most hybrids were registered from 1973 to 2022, though two of them are new creations and are currently being tested for their registration in the official catalog of cultivated plant varieties in Romania. Of the hybrids, 17 are single-cross hybrids, 14 are three-way-cross hybrids, and 4 are double-cross hybrids. According to the vegetation period, 16 are early and 19 are semi-early. Biological material was chosen taking into account the vegetation period, the period in which they were created, and the plant ideotype in order to identify the genotypes that cope best with unfavorable conditions.

### 4.2. Cultivation Technology and Experimental Design

Testing was carried out in the maize breeding fields of ARDS Turda, Cluj County, Romania (longitude 23°48′ E, latitude 46°35′ N). The area is hilly with different exposures and slopes. According to analyses performed by the Office for Pedological and Agrochemical Studies, Cluj-Napoca, the dominated soils are vertical clay–illuvial chernozem, with about 56% clay and 0.7% coarse sand, and the reaction is neutral (6.2–6.8 pH). Determined at a depth of 0–30 cm, the values of total nitrogen, phosphorus availability, potassium availability, carbonates, and humus are 0.2%, 65 ppm, 400 ppm, 0.7%, and 3.5%, respectively. A chemical analysis of the soil is carried out every 4 years. The values are averages, as they did not change significantly over the tested years.

The hybrids were studied in comparative plots laid out according to the randomized blocks method in three replicates, with each hybrid being sown in four rows of 5 m each (the middle ones being used for observations and yield quantification) (7mp) at a density of 70,000 plants/ha. Sowing was carried out with the Monoseed DT seeder for experimental plots from Wintersteiger Austria on 26 April (2019 and 2020), 1 May (2018 and 2023), and 4 May (2017, 2021, and 2022). In the experimental field, a three-year rotation (soya–winter wheat–maize) is used. Plowing and superficial tillage (before fertilizer and herbicide application) were carried out in autumn and spring, respectively. The soil was fertilized with NPK 27:13.5:0 complex fertilizers (400 kg ha^−1^) from Azomureș Romania, an Ameropa Company. The soil did not lack micro- or macronutrients. A reduced level of fertilization was used to highlight the yield capacity and adaptability of the genotype, not the crop technology. Only hybrids that are competitive under these conditions were promoted further. Two herbicides were applied as follows: 1.2 L/ha a.s. dimethenamid-p (720 g/L) at pre-emergence (Spectrum, produced by BASF, Ludwigshafen, Germany) and 2 L/ha a.s. tembotrione (44 g/L) and isoxadiphen-ethyl (22 g/L) at post-emergence (Laudis 66 OD, produced by Bayer AG, Crop Science Division, Monheim am Rhein, Germany).

### 4.3. Meteorological Data

**Figure 9:**
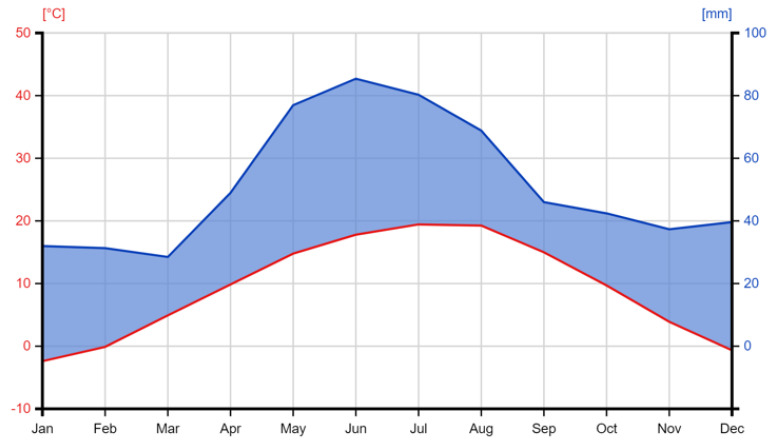
Long-term climatological characteristics of the studied area: average temperature (°C) and average precipitation (mm) (1957–2017) [72].

The meteorological data used were obtained from Turda Meteorological Station, located near the experimental fields. Temperatures and precipitation were compared to the 60-year averages (1957–2017), which represent the period from the establishment of ARDS Turda to the beginning of this study. According to the weather data, the warmest month is July, with an average temperature of 19.7 °C, followed by August with an average temperature of 19.3 °C. Precipitation values are between 18.8 mm (February) and 84.8 mm (June) (Figure 9).

### 4.4. Data Analysis

Data were analyzed using Past4 Soft, and Fisher’s protected least significant difference (LSD) test was used to determine the significance of the differences between genotypes and years and their interaction. The yield stability was analyzed based on the method of Eberhart and Russell (1966) [73], with the environmental indices calculated as the difference between the mean of all hybrids in each year and the overall mean (all hybrids in all years).

$$I_{\dot{j}}=\Sigma Y_{i..j}-\mu$$

where *I_j_* = environmental indices; *Y_i_…_j_* = the mean of all hybrids (*i*…) in the j environment (year); and *μ* = the overall mean.

## 5. Conclusions

The average number of days between sowing and flowering varied greatly both between experimental years and between hybrids or was influenced by the hybrid x year interaction. For all hybrids, the highest number of days between sowing and flowering was noted in 2020, the year with the fewest hours of sunshine and the lowest number of GDDs in the months of May and June. The fewest days to reach BBCH65 were needed in 2018 or 2022, depending on the hybrid.

New hybrids (Turda 332, Turda 344, Turda 335, Turda 2020, Turda 380, SUR18-399, and HST148) have a low ASI regardless of the climatic conditions, while the older ones are more prone to protandry. The highest ASI values and the most hybrids with protandry were observed in 2017 and 2022.

For both early and semi-early hybrids, the lowest yields were obtained in 2022 and 2023. For early hybrids, the highest yields were achieved in 2018, followed by 2020, while, for semi-early hybrids, 2021 was by far the most favorable year.

The new hybrids exceeded the experimental average by up to 33%, while the yield of old hybrids was up to 22% lower. The results suggest a better adaptation of the semi-early hybrids to the cultivation conditions in the area. The Turda344, Turda335, Turda 2020, Turda380, HST148, and SUR18-399 hybrids can be successfully cultivated in Transylvania on the Moldavian Plateau or in other areas with similar conditions. Early hybrids can be an alternative for secondary crops.

## Figures and Tables

**Table 1:** Maximum temperatures and the amount of rainfall in July.

Year	Maximum Temperature Range (°C)	Number of Rainy Days	Rainfall Sum/Day Range (mm)	Total Sum of Rainfall/Month (mm)
2017	20.7–32.3	16	0.2–24.2	110.2
2018	19.5–29.9	15	0.2–31.5	85.7
2019	19.2–34.7	10	0.2–15.2	35.0
2020	21.2–31.2	17	0.2–12.6	86.8
2021	21.7–33.7	8	0.4–36.5	123.1
2022	22.8–38.2	8	0.2–21.3	25.2
2023	26.0–34.2	15	0.2–39.7	85.8

**Table 2:** Number of heat days and temperatures for Turda in the period 2017–2023.

Year	T_max_ ≥ ..	Month		
		June	July	August
2017	32 °C	1	2	12
	35 °C	-	-	4
2018	32 °C	-	-	1
	35 °C	-	-	-
2019	32 °C	3	3	9
	35 °C	-	-	-
2020	32 °C	-	-	3
	35 °C	-	-	-
2021	32 °C	3	11	5
	35 °C	-	-	-
2022	32 °C	5	16	10
	35 °C	1	6	1
2023	32 °C	-	2	13
	35 °C	-	-	2

**Table 3:** The number of sunshine hours, sum of thermal degrees above 10 °C and average number of days from sowing to flowering.

Year	Number of Sunshine Hours			Growing Degree Days Index (May + June)	Average Number of Days Between Sowing and Flowering
	May	June	July		
2017	193.8	253.8	225.6	497.6	66
2018	250.0	185.8	176.7	550.5	65
2019	131.1	267.7	206.0	464.9	78
2020	173.0	167.7	206.8	387.4	84
2021	173.7	233.4	245.4	421.9	71
2022	239.8	264.0	250.5	527.7	65
2023	180.9	174.9	244.7	436.0	69

**Table 4:** The influence of the year on the yield.

Experimental Years	Average Yield (kg ha^−1^)	Difference ± Average (kg ha^−1^)	
2017 to 2023 average	8793	control	
2017	7904	−889	^000^
2018	10,475	+1682	***
2019	8413	−380	^0^
2020	10,121	+1328	***
2021	10,895	+2102	***
2022	6209	−2584	^000^
2023	7433	−1360	^000^
		LSD (*p* < 0.05) = 302 LSD (*p* < 0.01) = 423 LSD (*p* < 0.001) = 598	

*** = significant at *p* < 0.001 probability levels, positive values; ^0^, ^000^ = significant at *p* < 0.01 and *p* < 0.001 probability levels, negative values.

**Table 5:** The influence of the “hybrid” factor on the yield.

No.	Hybrid Name	Average Yield (kg ha^−1^)	Difference ± Average (kg ha^−1^)	
	Hybrid average	8793	control	
1.	HD115	6841	−1952	^000^
2.	HS105	6857	−1935	^000^
3.	HD211	7640	−1152	^000^
4.	HS105A	7050	−1742	^000^
5.	Turda200	8000	−793	^000^
6.	Turda215	7920	−873	^000^
7.	Turda228	7278	−1514	^000^
8.	Turda100	6998	−1795	^000^
9.	Turda213	7729	−1064	^000^
10.	Turda199	7566	−1226	^000^
11.	Turda160	7690	−1103	^000^
12.	Turda260	8688	−104	ns
13.	ELAN	7081	−1712	^000^
14.	Doina	7983	−809	^000^
15.	Saturn	9339	+546	*
16.	Turda200 Plus	7809	−983	^000^
17.	Turda Super	8344	−448	^0^
18.	Turda—SU181	7716	−1077	^000^
19.	Turda—SU182	8667	−126	ns
20.	Turda—SU210	8157	−635	^00^
21.	Turda-Mold188	8069	−724	^00^
22.	Turda Favorit	9726	+933	***
23.	Turda 165	8216	−577	^0^
24.	Turda 201	8749	−43	ns
25.	Turda 145	8829	+35	ns
26.	Turda Star	9469	+676	**
27.	Turda248	10,052	+1259	***
28.	Marius TD	9650	+857	***
29.	Turda332	10,760	+1967	***
30.	Turda344	11,223	+2430	***
31.	Turda335	11,521	+2727	***
32.	Turda2020	11,548	+2755	***
33.	Turda380	11,688	+2895	***
34.	HST148	11,372	+2579	***
35.	SUR18/399	11,517	+2724	***
			LSD (*p* < 0.05) = 437 LSD (*p* < 0.01) = 577 LSD (*p* < 0.001) = 738	

*, **, and ***/^0^, ^00^, and ^000^ = significant at *p* < 0.05, *p* < 0.01, and *p* < 0.001 probability levels, respectively, positive/negative values; ns = not significant.

## Data Availability

The original contributions presented in this study are included in the article/Supplementary Material, and further inquiries can be directed to the corresponding author/s.

## Supplementary Materials

The following supporting information can be downloaded at https://www.mdpi.com/article/10.3390/plants13233322/s1, Table S1: The maize hybrids studied.
