# Supplementary material for: Influence of Changing Weather on Old and New Maize Hybrids: A Case Study in Romania
Source: Plants (Basel). 2024 Nov 27;13(23):3322. doi: 10.3390/plants13233322 (PMC11644749; doi:10.3390/plants13233322)
Supplement: Supplementary file 1 [file plants-13-03322-s001.zip › plants-3277194-supplementary.pdf]

**Table S1.** The maize hybrids studied

| No. | Hybrid name | Hybrid type | Maturity group | Registration year |
|---|---|---|---|---|
| 1. | HD 115 | DCH | early | 1973 |
| 2. | HS 105 | SCH | early | 1971 |
| 3. | HD 211 | DCH | semi-early | 1975 |
| 4. | HS 105A | SCH | early | 1976 |
| 5. | Turda 200 | DCH | early | 1976 |
| 6. | Turda 215 | TWH | semi-early | 1976 |
| 7. | Turda 228 | TWH | early | 1979 |
| 8. | Turda 100 | DCH | early | 1979 |
| 9. | Turda 213 | SCH | semi-early | 1984 |
| 10. | Turda 199 | TWH | early | 1984 |
| 11. | Turda 160 | SCH | early | 1990 |
| 12. | Turda 260 | SCH | semi-early | 1990 |
| 13. | Elan | TWH | early | 1992 |
| 14. | Doina | TWH | early | 1994 |
| 15. | Saturn | SCH | semi-early | 1994 |
| 16. | Turda 200 Plus | TWH | early | 1996 |
| 17. | Turda Super | TWH | semi-early | 1996 |
| 18. | Turda - SU 181 | SCH | early | 2000 |
| 19. | Turda - SU 182 | SCH | early | 2000 |
| 20. | Turda – SU 210 | TWH | semi-early | 2000 |
| 21. | Turda-Mold 188 | TWH | early | 2001 |
| 22. | Turda Favorit | SCH | semi-early | 2001 |
| 23. | Turda 165 | TWH | early | 2002 |
| 24. | Turda 201 | TWH | semi-early | 2002 |
| 25. | Turda 145 | TWH | early | 2004 |
| 26. | Turda Star | TWH | semi-early | 2005 |
| 27. | Turda 248 | SCH | semi-early | 2012 |
| 28. | Marius TD | SCH | semi-early | 2013 |
| 29. | Turda 332 | SCH | semi-early | 2014 |
| 30. | Turda 344 | TWH | semi-early | 2017 |
| 31. | Turda 335 | SCH | semi-early | 2021 |
| 32. | Turda 2020 | SCH | semi-early | 2021 |
| 33. | Turda 380 | SCH | semi-early | 2022 |
| 34. | HST 148 | SCH | semi-early | In testing |
| 35. | SUR 18/399 | SCH | semi-early | In testing |

SCH=single cross hybrid; TWH= three-way cross hybrid; DCH=double cross hybrid
